# Endoscopic Ultrasound-Guided Gallbladder Drainage Using Double-Pigtail Plastic Stents for Acute Cholecystitis in Patients with Malignant Biliary Obstruction

**DOI:** 10.3390/jcm15166329

**Published:** 2026-08-16

**Authors:** Keiki Nagai, Yusuke Kurita, Yuji Fujita, Kensuke Kubota, Tomoki Ogata, Etsuko Yamabe, Hiroki Uechi, Yuji Koyama, Shintaro Tsujikawa, Yu Honda, Takayuki Oda, Takeshi Iizuka, Shin Yagi, Eisuke Suzuki, Seitaro Tsujino, Ken Ishii, Sho Hasegawa, Shingo Kato, Masato Yoneda

**Affiliations:** 1 Department of Gastroenterology and Hepatology, Yokohama City University Hospital, Yokohama, Kanagawa 236-0004, Japan; chococake1106@gmail.com (K.N.); kubotak@yokohama-cu.ac.jp (K.K.); tomo1993baske@gmail.com (T.O.); honda.yu.cy@yokohama-cu.ac.jp (Y.H.); taka-northbay@hotmail.co.jp (T.O.); iizuka.tak.ax@yokohama-cu.ac.jp (T.I.); t216074f@yokohama-cu.ac.jp (S.Y.); 17eisuke@gmail.com (E.S.); e073037g@gmail.com (S.T.); kenishii@ymail.ne.jp (K.I.); t166064d@yokohama-cu.ac.jp (S.H.); shin800m@yokohama-cu.ac.jp (S.K.); yoneda@yokohama-cu.ac.jp (M.Y.); 2 Department of Hepato-Biliary-Pancreatic Medicine, NTT Medical Center Tokyo, Tokyo 141-8625, Japan; yufuji5395@gmail.com (Y.F.); etsuko.19970506@gmail.com (E.Y.); uechi.h.3526@gmail.com (H.U.); k-yuji0909@nms.ac.jp (Y.K.); shintaro0717@gmail.com (S.T.)

**Keywords:** acute cholecystitis, cholecystostomy, cholestasis, extrahepatic, endosonography, stents

## Abstract

**Background/Objectives**: Acute cholecystitis in patients with malignant biliary obstruction (MBO) is challenging to manage. Evidence for endoscopic ultrasound-guided gallbladder drainage (EUS-GBD) using double-pigtail plastic stents (DPPS) in this setting remains limited. We evaluated the efficacy and safety of EUS-GBD using DPPS for acute cholecystitis in patients with MBO. **Methods**: We retrospectively reviewed 41 consecutive patients who underwent EUS-GBD using DPPS at two centers. The primary endpoint was technical success; secondary endpoints included clinical success, adverse events, recurrence, and subsequent oncologic treatment. **Results**: Technical success was achieved in 95.1% (39/41), with clinical success in all technically successful cases. Adverse events included biliary peritonitis (14.6%) and procedure-related DPPS migration (2.4%). Recurrence occurred in 2.6% of patients. Systemic therapy was resumed in 10 of 18 patients (55.6%) at a median of 15 days after EUS-GBD. **Conclusions**: EUS-GBD using DPPS achieved high technical and clinical success rates with a low recurrence rate in selected patients with acute cholecystitis and MBO. Systemic therapy could be resumed in a subset of patients following successful drainage. These findings suggest that DPPS-based EUS-GBD may be a feasible internal drainage option in this setting, although prospective comparative studies are needed to further establish its safety and effectiveness.

## 1. Introduction

Acute cholecystitis in patients with malignant biliary obstruction (MBO) is challenging to manage [1], as many patients with advanced pancreatic or biliary tract cancer are poor candidates for laparoscopic cholecystectomy [2]. In these patients, acute cholecystitis frequently develops after self-expandable metal stent (SEMS) placement because of tumor invasion of or mechanical obstruction of the cystic duct orifice by the stent itself, with a reported incidence of approximately 5–8% [3,4]. Such episodes may delay or preclude systemic therapy and adversely affect both prognosis and quality of life (QOL) [4]. Therefore, gallbladder drainage is recommended for patients who are not suitable for early cholecystectomy [1]. However, PTGBD requires an external fistula; therefore, the American Society for Gastrointestinal Endoscopy (ASGE) recommends endoscopic ultrasound-guided gallbladder drainage (EUS-GBD) over PTGBD for patients who cannot undergo cholecystectomy [5]. Furthermore, ETGBD may be technically challenging in cases with tumor invasion or altered anatomy [6]. EUS-GBD is a minimally invasive internal drainage technique for patients in whom surgery, PTGBD, or ETGBD is unsuitable [7]. Consequently, EUS-GBD has evolved considerably, particularly after the introduction of lumen-apposing metal stent (LAMS), which is now widely used [8,9].

EUS-GBD using a double-pigtail plastic stent (DPPS) has primarily been reported in benign gallbladder diseases or in poor surgical candidates [10]. Although several studies have demonstrated the feasibility of DPPS-based EUS-GBD for acute cholecystitis in patients with malignancy [11,12], evidence remains limited regarding its clinical outcomes and safety in patients with MBO, particularly with respect to recurrence and subsequent oncologic management. For acute cholecystitis in patients with MBO, the comparative role of DPPS versus LAMS remains unclear. Furthermore, whether DPPS-based internal drainage can provide sustained control of cholecystitis and facilitate subsequent oncologic treatment in patients with MBO has not been well established. Because acute cholecystitis in patients with MBO may interrupt systemic therapy and adversely affect prognosis and QOL, further evaluation of this treatment strategy is warranted. Therefore, we aimed to comprehensively evaluate technical and clinical success, safety, recurrence, and subsequent oncologic treatment outcomes, including resumption of systemic therapy and conversion surgery, after EUS-GBD using DPPS for acute cholecystitis in patients with MBO.

## 2. Materials and Methods

### 2.1. Patients

In this retrospective two-center study, we reviewed cases of EUS-GBD for acute cholecystitis in patients with MBO at Yokohama City University Hospital and NTT Medical Center Tokyo between November 2015 and December 2024. Patients with acute calculous cholecystitis considered primarily attributable to gallstones were not included in the study population. EUS-GBD was generally indicated for patients at high surgical risk, with a Charlson Comorbidity Index ≥ 6 [13] or an American Society of Anesthesiologists Physical Status classification ≥ 3 used as reference criteria. Surgical candidacy was ultimately determined by the treating physicians and surgeons based on a comprehensive assessment of the patient’s general condition and oncologic status, provided that the patient’s condition allowed sedation. Among patients requiring gallbladder drainage, the choice between EUS-GBD, PTGBD, and ETGBD was not based on predefined allocation criteria but was determined according to institutional practice, the patient’s clinical and anatomical conditions, and the discretion of the treating physician. Patients with suspected gallbladder perforation were excluded. The Institutional Review Board of Yokohama City University Hospital approved the study (approval no. B200600003), which was conducted in accordance with the Declaration of Helsinki. Only routine clinical data were used, patient confidentiality was protected, and opt-out consent was applied; patients who refused participation were excluded. The presumed etiology of cystic duct obstruction was retrospectively assessed based on available imaging findings and clinical information. No patients underwent PTGBD or PTGBA before EUS-GBD in this cohort.

### 2.2. EUS-GBD Procedure

**Figure 1:**
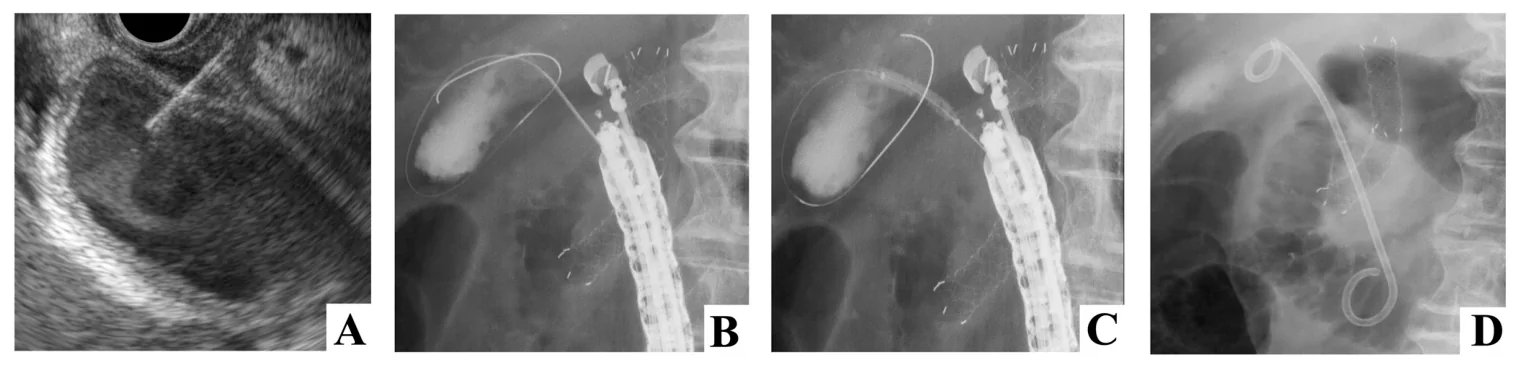
Endoscopic ultrasound-guided gallbladder drainage procedure. (**A**) The gallbladder is visualized from the duodenal bulb under endoscopic ultrasound guidance. After confirming a clear image, transduodenal puncture is performed using a 19-gauge needle (EZ Shot 3 Plus; Olympus, Tokyo, Japan). (**B**) After bile aspiration, cholecystography is performed to delineate the gallbladder cavity, and a 0.025-inch guidewire (VisiGlide2; Olympus) is advanced and coiled within the gallbladder. (**C**) The puncture tract is dilated using a 4 mm balloon catheter (REN; Kaneka, Osaka, Japan). (**D**) A 7-Fr double-pigtail plastic stent (10 cm; Gadelius Medical, Tokyo, Japan) is placed between the gallbladder and the duodenal bulb to establish internal drainage.

In all patients, EUS-GBD was performed under fluoroscopic guidance using a linear echoendoscope (GF-UCT260; Olympus Corp., Tokyo, Japan). All procedures were performed with the patient in the prone position under intravenous sedation by endoscopists experienced in EUS-guided tissue acquisition (EUS-TA) and therapeutic EUS, including EUS-GBD. The procedural steps are illustrated in Figure 1. Periprocedural antibiotic prophylaxis (typically a third-generation cephalosporin or equivalent broad-spectrum agent) was administered to all patients before puncture, and continued after the procedure according to the patient’s clinical course. CO_2_ insufflation was used routinely throughout the procedure. The gallbladder was visualized from the duodenal bulb, and after confirming clear delineation, it was punctured transduodenally using either a 19- or 22-gauge needle (EZ Shot 3 Plus; Olympus Corp., Tokyo, Japan). The needle gauge was selected at the operator’s discretion. After aspiration of a small amount of bile to confirm intragallbladder access, cholecystography was performed to delineate the gallbladder cavity. Subsequently, a 0.025-inch guidewire (VisiGlide2; Olympus Corp., Tokyo, Japan) or a 0.018-inch guidewire (Fielder18; ASAHI INTECC Co., Ltd., Seto, Aichi, Japan) was advanced into the gallbladder, with an adequate length of wire secured in the lumen. Tract dilation was then performed using either a 6-Fr or 7-Fr dilator (ES Dilator; Zeon Medical Inc., Tokyo, Japan), or a 4 mm balloon dilator (REN Balloon Catheter; Kaneka Corp., Osaka, Japan), selected according to the individual case at the operator’s discretion. Dilation was performed stepwise to create a sufficient fistulous tract between the duodenal wall and gallbladder. Subsequently, a DPPS (7 Fr, 7 or 10 cm; Gadelius Medical Co., Ltd., Tokyo, Japan; or Boston Scientific Corp., Marlborough, MA, USA) was deployed, with the proximal end positioned in the duodenal bulb. After confirming proper stent deployment, bile drainage from the gallbladder into the duodenal bulb was verified endoscopically, and the procedure was completed. Stent position was confirmed endoscopically or fluoroscopically during and immediately after the procedure to ensure the absence of bleeding or stent migration from the puncture site. All patients were hospitalized after the procedure for observation. Scheduled stent removal or routine surveillance endoscopy was not included in the routine management protocol because long-term or permanent stent indwelling was anticipated after EUS-GBD for acute cholecystitis in patients with MBO. Additional endoscopic evaluation or intervention was considered only when clinically indicated, such as in cases of suspected recurrent cholecystitis or stent-related complications.

### 2.3. Definitions

The diagnosis and severity grading of acute cholecystitis were determined according to the Tokyo Guidelines 2018 [1]. Biliary peritonitis was defined as new-onset or worsening abdominal pain with peritoneal signs after EUS-GBD [14]. Technical success was defined as successful deployment of a 7-Fr DPPS into the gallbladder with confirmation of internal bile drainage. Clinical success was defined as resolution of symptoms and signs, reduction in body temperature to <37.5 °C, and either a reduction in leukocyte count of at least 25% from baseline or a leukocyte count <10,000/μL within 3 days after EUS-GBD, without the need for additional gallbladder drainage or surgical intervention [15]. Follow-up CT and EUS for assessing resolution of acute cholecystitis were not routinely performed at either participating institution; therefore, imaging-based resolution criteria were not incorporated into this definition. Patients who did not meet all three criteria, or who required additional intervention during the observation window, were classified as clinical failures. Adverse events were classified according to the AGREE classification [16] and categorized as early (≤14 days) or late (>14 days). The denominator for adverse-event analyses was all intended procedures (n = 41). Recurrent acute cholecystitis was defined as the reappearance of TG18-compatible signs and symptoms of acute cholecystitis after index clinical success, requiring antibiotic therapy and/or additional intervention, irrespective of the underlying mechanism. Recurrent acute cholecystitis was evaluated only in patients who underwent successful DPPS placement (n = 39). Time to systemic therapy resumption was defined as the interval between EUS-GBD and the first administration of systemic therapy after clinical improvement.

### 2.4. Endpoints

The primary endpoint was technical success. Secondary endpoints included clinical success, procedure-related adverse events, recurrent acute cholecystitis, resumption of systemic therapy, time to systemic therapy resumption, and subsequent conversion surgery.

### 2.5. Statistical Analysis

Continuous variables were summarized as median (interquartile range [IQR]), and categorical variables were expressed as numbers and percentages. Exact 95% confidence intervals (CIs) for technical success, clinical success, adverse events, recurrence, and systemic therapy resumption were calculated using the exact Clopper–Pearson method. Continuous variables were compared between two groups using the Mann–Whitney U test. A two-sided *p* value < 0.05 was considered statistically significant. All statistical analyses were performed using SPSS version 29.0.1.0 (171) (IBM Corp., Armonk, NY, USA).

## 3. Results

### 3.1. Patient Characteristics

**Figure 2:**
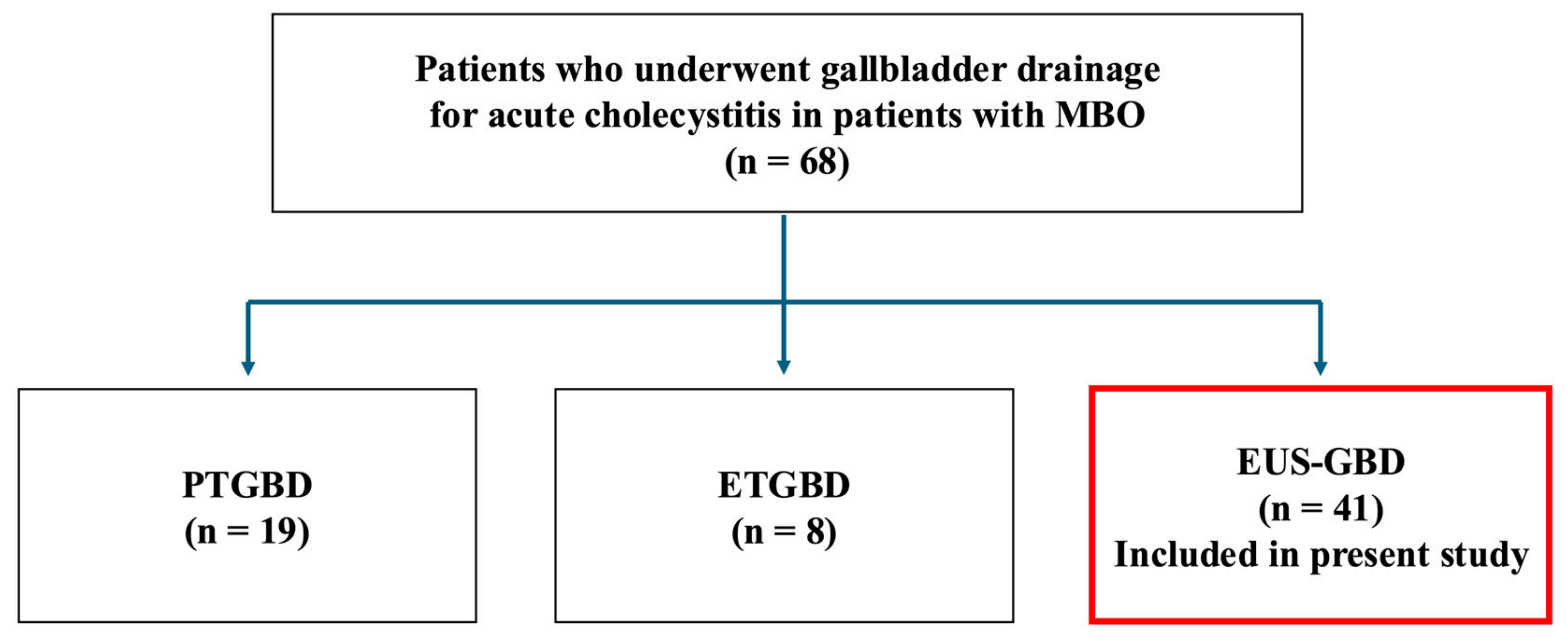
Flow diagram of patient selection. ETGBD, endoscopic transpapillary gallbladder drainage; EUS-GBD, endoscopic ultrasound-guided gallbladder drainage; MBO, malignant biliary obstruction; PTGBD, percutaneous transhepatic gallbladder drainage.

During the study period, 68 patients underwent gallbladder drainage for acute cholecystitis in patients with MBO. Of these, 19 underwent PTGBD, 8 underwent ETGBD, and 41 underwent EUS-GBD using DPPS. The 41 patients who underwent EUS-GBD using DPPS were included in the present study (Figure 2). Of these, 25 patients were treated at NTT Medical Center Tokyo and 16 at Yokohama City University Hospital.

The baseline characteristics of the study population are summarized in Table 1. The median age was 80 years (IQR, 73–86), and 21 patients (51.2%) were male. The median Charlson Comorbidity Index was 7 (IQR, 6–8). ECOG performance status was 0 in 3 patients (7.3%), 1 in 13 (31.7%), 2 in 17 (41.5%), 3 in 6 (14.6%), and 4 in 2 (4.9%). At the onset of acute cholecystitis, 18 patients (43.9%) were receiving systemic therapy. Primary diseases included pancreatic cancer (n = 17), cholangiocarcinoma (n = 17), colon cancer (n = 2), and gallbladder cancer, rectal cancer, duodenal cancer, gastric neuroendocrine tumor, and hepatocellular carcinoma (n = 1 each). Cancer stage was II in 2 patients (4.9%), III in 14 (34.1%), and IV in 25 (61.0%). According to the TG18 classification, acute cholecystitis was graded as mild in 8 patients (19.5%), moderate in 27 (65.9%), and severe in 6 (14.6%). Previously placed biliary stents included self-expandable metal stents in 23 patients (56.1%), plastic stents in 13 (31.7%), and no biliary stent in 5 (12.2%). Among patients with previously placed biliary stents, the median interval from biliary stent placement to acute cholecystitis was 35 days (IQR, 12–60). The presumed etiology of cystic duct obstruction was a self-expandable metal stent in 23 patients (56.1%), a plastic stent in 13 (31.7%), lymph node metastasis in 3 (7.3%), peritoneal dissemination in 1 (2.4%), and gallbladder cancer in 1 (2.4%).

### 3.2. Procedural Outcomes

Procedural details are presented in Table 2. The technical success rate of EUS-GBD was 95.1% (39/41). A 19-gauge needle was used in 40 patients, whereas a 22-gauge needle was used in one patient. A 0.025-inch guidewire was used with the 19-gauge needle, whereas a 0.018-inch guidewire was used with the 22-gauge needle. Tract dilation was performed using a mechanical dilator alone in eight patients, both mechanical and balloon dilators in 14 patients, and a balloon dilator alone in 19 patients. A 7-Fr, 10 cm stent was used in 37 patients, whereas a 7-Fr, 7 cm stent was used in two patients. Technical failure occurred in two patients, both of whom subsequently underwent rescue endoscopic nasobiliary drainage (ENBD). The median procedure time was 25 min (IQR, 17–35).

In the two patients with technical failure, the gallbladder was successfully punctured using a 19-gauge needle, and tract dilation was completed with a balloon dilator. However, the 7-Fr DPPS could not be advanced through the gallbladder wall. Therefore, a 5-Fr ENBD tube was placed as rescue drainage. After resolution of acute cholecystitis, the ENBD tube was transected within the stomach using a loop cutter to achieve internal drainage. Neither patient experienced recurrent acute cholecystitis during follow-up.

### 3.3. Clinical and Follow-Up Outcomes

Clinical and follow-up outcomes are summarized in Table 3. Clinical success was achieved in all technically successful cases (39/39; 100%; 95% CI, 91.0–100.0). The median time to abdominal pain resolution was 1 day (IQR, 1–2), and the median time to resumption of oral intake was 2 days (IQR, 1–2). The median C-reactive protein level decreased from 13.9 mg/dL (IQR, 6.05–20.2) at baseline to 2.1 mg/dL (IQR, 1.2–3.8) at day 7 after EUS-GBD, while the median leukocyte count decreased from 8200/μL (IQR, 6300–12,000) to 5800/μL (IQR, 4400–7000).

Procedure-related adverse events occurred in seven patients (17.1%). Biliary peritonitis developed in six patients (14.6%; 95% CI, 5.6–29.2), with all cases occurring on the day after EUS-GBD. All six cases were classified as AGREE grade II and were managed conservatively with fasting and intravenous antibiotic therapy, without requiring additional drainage or surgical intervention. Procedure-related DPPS migration occurred in one patient (2.4%; 95% CI, 0.1–12.9). The patient developed acute cholangitis 56 days after EUS-GBD. During endoscopic retrograde cholangiopancreatography performed for biliary stent placement, the DPPS was dislodged through contact with the endoscope. Cholecystitis recurred 7 days later, and repeat EUS-GBD with placement of a new 7-Fr DPPS was successfully performed. No further recurrence occurred during follow-up.

The median length of hospital stay was 13 days (IQR, 9–19). Patients with biliary peritonitis had a median hospital stay of 19 days (IQR, 14.5–20.5), compared with 12 days (IQR, 8.5–17.0) in those without biliary peritonitis (*p* = 0.222). The median follow-up period was 105 days (IQR, 59–402). Recurrent acute cholecystitis occurred in one patient (2.6%; 95% CI, 0.1–13.5). No procedure-related deaths were observed.

### 3.4. Resumption of Systemic Therapy

Details of the patients receiving systemic therapy are presented in Table 4. Among the 39 patients who underwent technically successful EUS-GBD with DPPS placement, 18 were receiving systemic therapy for their primary malignancy at the onset of acute cholecystitis. Following resolution of acute cholecystitis and improvement in their general condition, systemic therapy was resumed in 10 patients (55.6%; 95% CI, 30.8–78.5). The median time to systemic therapy resumption was 15 days (IQR, 12–20). Among the remaining eight patients, systemic therapy was not resumed because of deterioration in performance status (n = 5), disease progression (n = 2), or patient preference (n = 1).

One patient with hilar cholangiocarcinoma successfully resumed systemic therapy and subsequently underwent conversion surgery consisting of right hepatectomy after adequate control of acute cholecystitis.

## 4. Discussion

In this study, the technical success rate of EUS-GBD using a 7-Fr DPPS was 95.1%, and clinical success was achieved in all technically successful cases. The recurrence rate of acute cholecystitis was low (2.6%), and systemic therapy was resumed in 10 of 18 patients who had received systemic therapy at the onset of cholecystitis. In addition, one patient subsequently underwent conversion surgery after successful control of acute cholecystitis. However, biliary peritonitis occurred in six patients (14.6%), highlighting the need for careful consideration of procedure-related adverse events. Overall, these findings suggest that EUS-GBD using DPPS may represent a feasible internal drainage option for selected patients with acute cholecystitis and MBO, although its safety requires further evaluation.

Although direct comparisons across studies should be interpreted cautiously, the technical success observed in the present study fell within the range of previously reported outcomes for PTGBD (98.1%) [17], ETGBD (83.0%) [18], LAMS (94.0%) [8], and SEMS (98.4%) [19]. In the two cases of technical failure, a 7-Fr DPPS could not be advanced through the gallbladder wall, and potential contributing factors included a thickened gallbladder wall, poor adhesion to the adjacent gastrointestinal tract, and excessive mobility of the gallbladder [7]. The clinical success rate was 100% among the technically successful cases, which was also within the range reported for PTGBD (95.6%) [17], ETGBD (88.1%) [18], SEMS (98.4%) [19], and LAMS (96.0%) [20]. However, because this study was a retrospective, single-arm investigation without a control group, it does not establish the superiority or non-inferiority of DPPS over SEMS or LAMS. Furthermore, comparisons across studies should be interpreted with caution because differences in patient selection, indications, disease severity, and outcome definitions may substantially influence the reported results, irrespective of stent type. The importance of appropriate indications and patient selection in interpreting the outcomes of therapeutic EUS procedures has also been emphasized in the context of EUS-guided biliary drainage [21]. Taken together, these findings suggest that DPPS may represent a feasible internal drainage option in appropriately selected patients.

The overall adverse event rate of EUS-GBD using DPPS was 17.1%. Biliary peritonitis was the most frequent adverse event, occurring in six patients (14.6%); all cases were classified as AGREE grade II and resolved with conservative management without the need for additional drainage or surgery. Despite successful conservative management in all cases, biliary peritonitis tended to be associated with prolonged hospitalization. Although the rate of biliary peritonitis in the present study appeared relatively high, bile leakage is a recognized concern with plastic stent-based EUS-GBD. SEMS may provide better sealing of the gap between the stent and the puncture tract than plastic stents, thereby reducing the risk of bile leakage [22]. In contrast, DPPS may be associated with a risk of bile leakage because of its small diameter and lack of a lumen-apposing feature. In addition, the method of fistula dilation may influence the risk of bile leakage [23]. The reported incidence of bile leakage and biliary peritonitis with EUS-GBD using plastic stents is approximately 14% [24], which is consistent with the 14.6% observed in the present study. These findings suggest that minimizing bile leakage during tract dilation and stent deployment may be particularly important when DPPS is used. As a potential strategy to reduce bile leakage, a technique has been reported in which the inner sheath of the delivery system is partially withdrawn before DPPS deployment, allowing aspiration and irrigation of gallbladder contents through the DPPS system [25].

Stent migration represents another potential stent-related adverse event. In the present study, one patient experienced procedure-related DPPS migration during ERCP for biliary stent placement rather than spontaneous migration. Repeat EUS-GBD with DPPS replacement was successfully performed, and no further recurrence occurred. Although the type of stent and clinical context differed from those in the present case, endoscopic management of a completely intra-gallbladder migrated LAMS through a secondary EUS-GBD has recently been reported [26]. This report, together with our experience, suggests that selected stent-related adverse events after EUS-GBD may be amenable to endoscopic reintervention.

The recurrence rate of cholecystitis after EUS-GBD using DPPS was 2.6%. Harada et al. reported a recurrence rate of 13.3% in patients with malignancy-associated cholecystitis undergoing EUS-GBD using plastic stents and identified malignancy as a significant risk factor for recurrence [11]. Despite including exclusively patients with acute cholecystitis and MBO, recurrence occurred in only one patient in the present study. These findings suggest that DPPS-based EUS-GBD may provide sustained internal drainage in selected patients with malignancy-associated cholecystitis, although the small sample size and limited number of recurrence events warrant cautious interpretation.

Among the 18 patients who were receiving systemic therapy at the onset of cholecystitis, 10 were able to resume systemic therapy after EUS-GBD, with a median time to resumption of 15 days (IQR, 12–20). Although few studies have systematically evaluated systemic therapy resumption after EUS-GBD using DPPS for acute cholecystitis in patients with MBO, a small case series using LAMS also reported resumption or initiation of systemic therapy within approximately 2 weeks [27]. Because PTGBD requires management of an external drainage tube and may be associated with recurrent cholecystitis and the need for repeat intervention [28], internal drainage via EUS-GBD avoids the need for external tube management and may be advantageous in selected patients in whom continuation of systemic therapy is anticipated. However, because the present study lacked a comparator group, whether EUS-GBD increases the likelihood of systemic therapy resumption or shortens the time to resumption compared with other drainage strategies cannot be determined. DPPS-based EUS-GBD has also been reported to permit subsequent conversion surgery in selected patients [29], and in the present study, one patient proceeded to curative surgery after resolution of acute inflammation.

This study has several limitations. First, as a retrospective study, patient selection and treatment strategies were left to the discretion of each institution, and the choice of EUS-GBD was not based on predefined criteria, raising the possibility of selection bias. Second, due to insurance coverage restrictions in Japan during the study period, LAMS could not be used, and DPPS was used in all cases. In addition, patients with suspected gallbladder perforation were excluded. Although recent multicenter evidence suggests that EUS-GBD using LAMS may be feasible in selected patients with contained gallbladder perforation, the applicability of our findings to this population remains uncertain [30]. Third, because this was a single-arm study without comparator groups, including SEMS, LAMS, or PTGBD, direct comparisons with other drainage methods cannot be made, and the relative efficacy and safety of DPPS could not be determined. Fourth, the sample size was relatively small, limiting the precision of the estimated rates of adverse events and recurrence and precluding meaningful subgroup analyses.

## 5. Conclusions

EUS-GBD using DPPS achieved high technical and clinical success rates with a low recurrence rate during follow-up in selected patients with acute cholecystitis and MBO. However, biliary peritonitis occurred in 14.6% of patients, highlighting the need for careful consideration of procedure-related safety. These findings should be interpreted in the context of the retrospective, single-arm design and limited sample size. Prospective comparative studies are warranted to further establish the safety and effectiveness of DPPS-based EUS-GBD in this population.

## Figures and Tables

**Table 1:** Patient characteristics.

Characteristic	Total (n = 41)
Age, years, median (IQR)	80 (73–86)
Sex, male/female	21/20
**ECOG performance status, n (%)**	
0	3 (7.3)
1	13 (31.7)
2	17 (41.5)
3	6 (14.6)
4	2 (4.9)
**ASA-PS classification, n (%)**	
I	0
II	38 (92.7)
III	3 (7.3)
IV	0
Charlson Comorbidity Index, median (IQR)	7 (6–8)
Charlson Comorbidity Index ≥ 6, n (%)	38 (92.7)
Receiving systemic therapy at onset of acute cholecystitis, n (%)	18 (43.9)
**Primary disease, n (%)**	
Pancreatic cancer	17 (41.5)
Cholangiocarcinoma	17 (41.5)
Colon cancer	2 (4.9)
Gallbladder cancer	1 (2.4)
Rectal cancer	1 (2.4)
Duodenal cancer	1 (2.4)
Gastric neuroendocrine tumor	1 (2.4)
Hepatocellular carcinoma	1 (2.4)
Cancer stage, n (%) *	
Stage II	2 (4.9)
Stage III	14 (34.1)
Stage IV	25 (61.0)
**Severity of acute cholecystitis, n (%) ^†^**	
Mild	8 (19.5)
Moderate	27 (65.9)
Severe	6 (14.6)
**Previously placed biliary stent, n (%)**	
Self-expandable metal stent	23 (56.1)
Plastic stent	13 (31.7)
None	5 (12.2)
Time from biliary stent placement to acute cholecystitis, days, median (IQR) ^‡^	35 (12–60)
**Etiology of cystic duct obstruction, n (%)**	
Self-expandable metal stent	23 (56.1)
Plastic stent	13 (31.7)
Lymph node metastasis	3 (7.3)
Peritoneal dissemination	1 (2.4)
Gallbladder cancer	1 (2.4)

**Abbreviations:** ASA-PS, American Society of Anesthesiologists Physical Status; ECOG, Eastern Cooperative Oncology Group; IQR, interquartile range. * Cancer stage was determined according to the staging system applicable to each primary malignancy. ^†^ Acute cholecystitis severity was graded according to the Tokyo Guidelines 2018. ^‡^ Calculated among patients with a previously placed biliary stent (n = 36).

**Table 2:** Procedural outcomes of endoscopic ultrasound-guided gallbladder drainage.

Outcome	Total (n = 41)
Technical success, n (%)	39 (95.1; 95% CI, 83.5–99.4)
**Needle and guidewire used, n (%)**	
19-gauge needle with 0.025-inch guidewire	40 (97.6)
22-gauge needle with 0.018-inch guidewire	1 (2.4)
**Tract dilation method, n (%)**	
Balloon dilator only	19 (46.3)
Mechanical and balloon dilators	14 (34.1)
Mechanical dilator only	8 (19.5)
**Stent type, n (%)**	
Double-pigtail plastic stent	39 (95.1)
7-Fr, 10 cm	37 (90.2)
7-Fr, 7 cm	2 (4.9)
**Rescue drainage after technical failure, n (%)**	
5-Fr ENBD	2 (4.9)
Procedure time, min, median (IQR)	25 (17–35)

**Abbreviations:** CI, confidence interval; ENBD, endoscopic nasobiliary drainage; IQR, interquartile range.

**Table 3:** Clinical and follow-up outcomes of endoscopic ultrasound-guided gallbladder drainage.

Outcome	Total (n = 41)
Clinical success, n (%)	39 (100; 95% CI, 91.0–100.0)
Time to abdominal pain resolution, days, median (IQR)	1 (1–2)
Time to resumption of oral intake, days, median (IQR)	2 (1–2)
Baseline C-reactive protein, mg/dL, median (IQR)	13.9 (6.05–20.2)
Baseline leukocyte count, /μL, median (IQR)	8200 (6300–12,000)
C-reactive protein at day 7 after EUS-GBD, mg/dL, median (IQR)	2.1 (1.2–3.8)
Leukocyte count at day 7 after EUS-GBD, /μL, median (IQR)	5800 (4400–7000)
**Adverse events, n (%)**	
Biliary peritonitis	6 (14.6; 95% CI, 5.6–29.2)
Procedure-related DPPS migration	1 (2.4; 95% CI, 0.1–12.9)
Length of hospital stay, days, median (IQR)	13 (9–19)
Follow-up period, days, median (IQR)	105 (59–402)
Recurrent acute cholecystitis, n (%)	1 (2.6; 95% CI, 0.1–13.5)
Procedure-related mortality, n (%)	0 (0)

**Abbreviations:** CI, confidence interval; DPPS, double-pigtail plastic stent; EUS-GBD, endoscopic ultrasound-guided gallbladder drainage; IQR, interquartile range.

**Table 4:** Clinical characteristics and course of acute cholecystitis during systemic therapy.

Characteristic/Outcome	Total (n = 18)
Age, years, median (IQR)	77.5 (73–85)
**Sex, male/female**	9/9
**ECOG performance status, n (%)**	
0	1 (5.6)
1	9 (50.0)
2	7 (38.9)
3	0
4	1 (5.6)
**Primary disease, n (%)**	
Pancreatic cancer	12 (66.7)
Cholangiocarcinoma	4 (22.2)
Colon cancer	1 (5.6)
Gastric neuroendocrine tumor	1 (5.6)
Cancer stage, n (%) *	
Stage III	4 (22.2)
Stage IV	14 (77.8)
**Systemic therapy at the onset of acute cholecystitis, n (%)**	
Gemcitabine	4 (22.2)
Gemcitabine plus nab-paclitaxel	3 (16.7)
S-1	4 (22.2)
Gemcitabine plus cisplatin	1 (5.6)
Gemcitabine plus cisplatin plus S-1	1 (5.6)
Modified FOLFIRINOX	1 (5.6)
Nanoliposomal irinotecan plus 5-fluorouracil/leucovorin	2 (11.1)
Panitumumab	1 (5.6)
Peptide receptor radionuclide therapy	1 (5.6)
**Tumor response status at the onset of acute cholecystitis, n (%)**	
Stable disease (SD)	16 (88.9)
Progressive disease (PD)	2 (11.1)
Systemic therapy resumed, n (%)	10 (55.6; 95% CI, 30.8–78.5)
Time from EUS-GBD to systemic therapy resumption, days, median (IQR)	15 (12–20)
**Reason for not resuming systemic therapy (n = 8), n (%)**	
Deterioration in performance status	5 (62.5)
Disease progression	2 (25.0)
Patient preference	1 (12.5)
Conversion surgery after EUS-GBD, n (%)	1 (5.6)

**Abbreviations:** CI, confidence interval; ECOG, Eastern Cooperative Oncology Group; EUS-GBD, endoscopic ultrasound-guided gallbladder drainage; IQR, interquartile range; PD, progressive disease; SD, stable disease. * Cancer stage was determined according to the staging system applicable to each primary malignancy.

## Data Availability

The datasets generated and/or analyzed during the current study are available from the corresponding author upon reasonable request. These data are not publicly accessible because of ethical and privacy considerations.

## Abbreviations

The following abbreviations are used in this manuscript:

ASGE	American Society for Gastrointestinal Endoscopy
DPPS	Double-pigtail plastic stent
ENBD	Endoscopic nasobiliary drainage
EUS-GBD	Endoscopic ultrasound-guided gallbladder drainage
ETGBD	Endoscopic transpapillary gallbladder drainage
LAMS	Lumen-apposing metal stent
MBO	Malignant biliary obstruction
PTGBD	Percutaneous transhepatic gallbladder drainage
SEMS	Self-expandable metal stent
QOL	Quality of life
